# Development of a Novel Circulating Autoantibody Biomarker Panel for the Identification of Patients with ‘Actionable’ Pulmonary Nodules

**DOI:** 10.3390/cancers15082259

**Published:** 2023-04-12

**Authors:** Claire Auger, Hita Moudgalya, Matthew R. Neely, Jeremy T. Stephan, Imad Tarhoni, David Gerard, Sanjib Basu, Cristina L. Fhied, Ahmed Abdelkader, Moises Vargas, Shaohui Hu, Tyler Hulett, Michael J. Liptay, Palmi Shah, Christopher W. Seder, Jeffrey A. Borgia

**Affiliations:** 1 Department of Anatomy & Cell Biology, Rush University Medical Center, Chicago, IL 60612, USA; 2 Rush University Medical College, Rush University Medical Center, Chicago, IL 60612, USA; 3 Division of Medical Oncology, Rush University Medical Center, Chicago, IL 60612, USA; 4 CDI Laboratories, Mayagüez, PR 00680, USA; 5 Department of Cardiovascular and Thoracic Surgery, Rush University Medical Center, Chicago, IL 60612, USA; 6 Department of Diagnostic Radiology, Rush University Medical Center, Chicago, IL 60612, USA; 7 Department of Pathology, Rush University Medical Center, Chicago, IL 60612, USA

**Keywords:** screening, biomarkers, autoantibodies, low-dose computed tomography

## Abstract

**Simple Summary:**

Circulating biomarkers for the identification of patients with “actionable” nodules may increase screening uptake and decrease false-positive rates associated with low-dose computed tomography (LDCT). Novel autoantibody biomarkers were identified utilizing a HuProt™ protein microarray. Luminex assays were developed for the targeted measurement of identified biomarkers within a large Biomarker Development Cohort (*n* = 841). Each individual biomarker’s performance was assessed. The Biomarker Development Cohort was split into three separate cohorts: Training, Validation 1, and Validation 2. Utilizing a Training cohort, a random forest model for identifying patients with “actionable” nodules from those with “non-actionable” nodules was built. The random forest model performance characteristics were determined for both a Validation 1 and the Validation 2 cohort. From these steps we have developed a risk-stratification method that assesses circulating levels of a panel of novel autoantibody biomarkers to serve as a companion diagnostic method for lung cancer screening.

**Abstract:**

Due to poor compliance and uptake of LDCT screening among high-risk populations, lung cancer is often diagnosed in advanced stages where treatment is rarely curative. Based upon the American College of Radiology’s Lung Imaging and Reporting Data System (Lung-RADS) 80–90% of patients screened will have clinically “non-actionable” nodules (Lung-RADS 1 or 2), and those harboring larger, clinically “actionable” nodules (Lung-RADS 3 or 4) have a significantly greater risk of lung cancer. The development of a companion diagnostic method capable of identifying patients likely to have a clinically actionable nodule identified during LDCT is anticipated to improve accessibility and uptake of the paradigm and improve early detection rates. Using protein microarrays, we identified 501 circulating targets with differential immunoreactivities against cohorts characterized as possessing either actionable (*n =* 42) or non-actionable (*n =* 20) solid pulmonary nodules, per Lung-RADS guidelines. Quantitative assays were assembled on the Luminex platform for the 26 most promising targets. These assays were used to measure serum autoantibody levels in 841 patients, consisting of benign (BN; *n =* 101), early-stage non-small cell lung cancer (NSCLC; *n =* 245), other early-stage malignancies within the lung (*n =* 29), and individuals meeting United States Preventative Screening Task Force (USPSTF) screening inclusion criteria with both actionable (*n =* 87) and non-actionable radiologic findings (*n =* 379). These 841 patients were randomly split into three cohorts: Training, Validation 1, and Validation 2. Of the 26 candidate biomarkers tested, 17 differentiated patients with actionable nodules from those with non-actionable nodules. A random forest model consisting of six autoantibody (Annexin 2, DCD, MID1IP1, PNMA1, TAF10, ZNF696) biomarkers was developed to optimize our classification performance; it possessed a positive predictive value (PPV) of 61.4%/61.0% and negative predictive value (NPV) of 95.7%/83.9% against Validation cohorts 1 and 2, respectively. This panel may improve patient selection methods for lung cancer screening, serving to greatly reduce the futile screening rate while also improving accessibility to the paradigm for underserved populations.

## 1. Introduction

Lung cancer is the leading cause of cancer-related mortality, largely due to late diagnosis. One key contributor to the particularly poor outcomes seen in patients with NSCLC is that almost half of all NSCLC cases are not detected until after they are advanced [1,2]. While LDCT scans have a sensitivity of 93.7%, they are plagued with a high false positivity rate. Only 3.6% of the initial positive scans are eventually classified as lung malignancies [3,4]. LDCT is only offered to limited patients based on relatively strict eligibility criteria, specifically those who have a 20 pack–year smoking history, are between the ages of 50–80, and are either current smokers or those who have quit smoking within the last 15 years. Based on the smoking status alone, it is estimated that at least half of the patients diagnosed with lung cancer would not qualify for screening [3]. Compounding this issue, among those who are eligible for screening, compliance has been exceptionally low, estimated at 4–14% [5,6]. Screening rates can vary widely based on where a patient is resides and their socioeconomic status [5,7]. 

In the latest screening recommendation, the United States Preventative Task Force (USPSTF) noted a need for biomarkers that can identify patients at high-risk of developing lung cancer and lower the rate of false positives [5]. To this end, our laboratory identified circulating biomarkers that can be used as molecular indicators to identify individuals likely to have clinically “actionable” solid pulmonary nodules. Actionable nodules, based upon the Lung-RADS v1.1 and v2022 definition (Lung-RADS 3 or 4), consists of individuals with solid pulmonary nodules that are equal to or greater than 6 mm [8,9]. These patients are considered high risk for lung cancer, and it is recommended that they be screened more frequently or undergo additional testing, which is often invasive. One study found that approximately 80.6% of patients had clinically “non-actionable” nodules and a low risk of lung malignancy (<0.1%). This can be compared to a lung malignancy risk of 0.9% in patients with “indeterminate” nodules (≥6 mm, <8 mm, Lung-RADS 3), and 9.6% for those with positive LDCT (≥8 mm, Lung-RADS 4) [9]. With a biomarker test that can identify patient populations with actionable nodules, we can identify patients who would benefit most from LDCT scans. This method could potentially increase uptake of screening in underserved populations as blood-based tests are more readily accessible, serve to ameliorate those with screening hesitancy since the test could be administered in primary care, and help lower false-positive rates.

This study is focused on developing a panel of circulating autoantibody biomarkers to answer this clinical need. We begin with a discovery effort that uses high-throughput protein microarrays to identify candidate biomarkers with differential signal between patients with clinically actionable and non-actionable nodules. These candidate biomarkers are then assessed in tandem with other potential biomarkers for this application with custom Luminex assays built ‘in house’ with a larger cohort of patients. Machine learning was then used to identify the optimal combination of autoantibody biomarkers for discerning actionable and non-actionable nodules, which can serve as a companion risk-stratification method in conjunction with current lung cancer screening protocols. 

## 2. Materials and Methods

**Figure 1:**
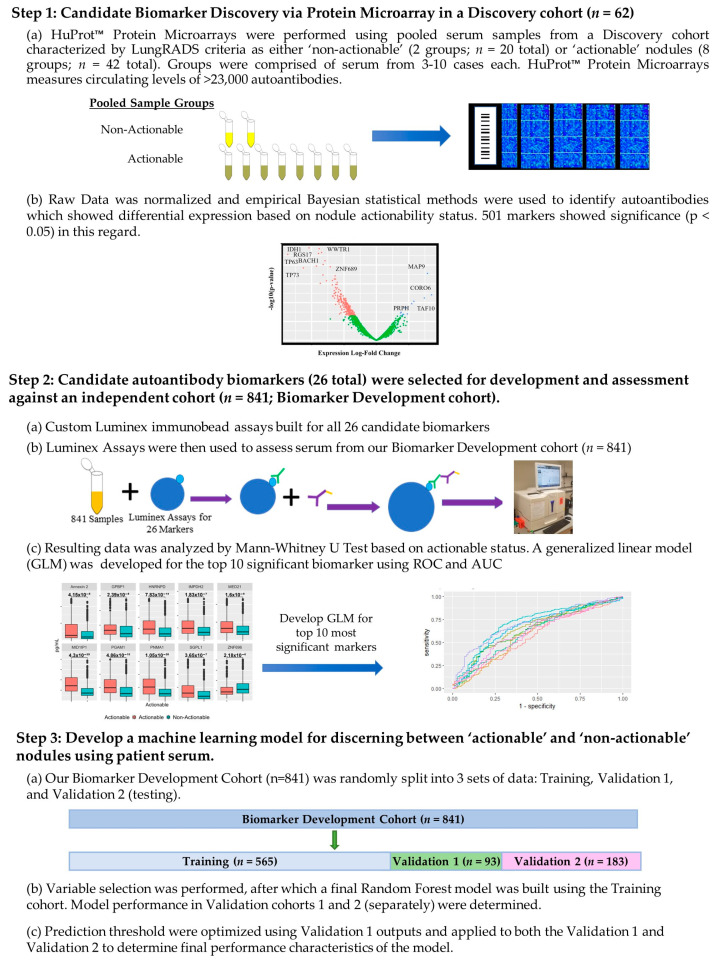
Experimental workflow. Illustration of all experiments and methodology with annotations. Step 1 involves the discovery of novel candidate biomarkers for discerning actionable versus non-actionable nodules utilizing HuProt™ microarrays with our “Discovery” cohort (*n =* 62 total samples; divided into 10 sample pools). A total of 26 candidate biomarkers were identified: 20 from microarray studies and 6 from our previous work. In Step 2, custom Luminex immunobead assays are developed for the candidate biomarkers identified in Step 1 and used to assess their performance against our Biomarker Development Cohort (*n =* 841). Each marker was statistically evaluated for its individual value for discerning actionable versus non-actionable nodules. In Step 3, the data from the Biomarker Development Cohort (*n =* 841) is split into three cohorts: Training, Validation 1, and Validation 2. The random forest algorithm was used to develop a biomarker panel based on the optimal combination of six features. The model’s performance characteristics for discerning actionable versus non-actionable cases were evaluated and optimized using Validation cohorts 1 and 2.

The overall methodology of this paper can be divided into three steps, which are laid out in Figure 1. These steps will be referred to throughout the Section 2.

### 2.1. Patient Cohorts

All serum samples were obtained from the Rush University Cancer Center Biorepository. This facility operated under full Rush IRB approval and enrolled all patients used in this study with written, informed consent prior to biospecimen collection. Whole blood samples were collected from all patients enrolled in this study, post LDCT screening but prior to surgical intervention or treatment. Whole blood was collected using red-top vacutainers by a trained phlebotomist. The blood was processed upon centrifugation per standard techniques and as reported previously [10]. The serum was stored aliquoted in a −80 °C freezer until being pulled for evaluation. All samples were collected between 2013–2019. Cases denoted as having lung malignancy or as benign were classified based on a pathological diagnosis of tissue obtained from anatomic resections. The high-risk screening cohort was comprised of patients who qualified for lung cancer screening based on USPSTF guidelines but did not have lung malignancy at the time of the blood draw. The patient samples included in this study are reflective of the “real world” population of patients who are screened or undergoing surgical resection for benign/malignant nodules at Rush University Medical Center in Chicago, IL. No other histological or molecular criteria were applied during patient selection, and therefore, the population assessed is effectively random.

Two separate cohorts were collected for the purposes of this study. The first is a Discovery cohort (*n =* 62) used in our experiments to identify novel candidate biomarkers via the HuProt microarray (Figure 1, Step 1). The second cohort we termed the Biomarker Development cohort (*n =* 841), as illustrated in Figure 1, Step 2. This cohort was randomly divided into Training (*n =* 565), Validation 1 (*n =* 93), and Validation 2 (*n =* 183; aka a ‘test’ cohort) cohorts to permit the development and validation of a multi-analyte panel for classifying patients based on actionable nodule status, as shown in Figure 1, Step 3.

Cases for this study were classified as actionable versus non-actionable based on nodule sizes annotated in the radiology report by a board-certified radiologist. All cases with solid nodules ≥6 mm were considered actionable, consistent with Lung-RADS v1.1 and v2022 [2,3]. For instances of nodules that fell between 4 mm to 6 mm, Lung-RADS scores were pulled from the radiologist reports and are reflective of the time of screening/sample collection. These Lung-RADS scores were based on either Lung-RADS v1.0 or v1.1, depending on the time of screening/specimen collection. Cases which were not classified as actionable were classified as non-actionable. 

### 2.2. High-Density Protein Microarrays (Illustrated in Step 1 of Figure 1)

Patient sera from the Discovery cohort were evaluated on high-density HuProt^TM^ v4.0 Protein Proteome microarrays by CDI Laboratories. HuProt^TM^ v4.0 Protein Proteome microarrays have been found to be a reproducible mechanism for discovery of autoantibody targets within malignancy samples and have been the basis of other studies exploring the development of machine learning models for malignancy prognosis and diagnosis [11,12]. A total of 10 microarrays were run on pooled samples for this study; the samples were then divided into ‘non-actionable’ and ‘actionable’ nodule categories. Non-actionable nodules were assessed as two groups (*n =* 10 per group), whereas actionable nodules were assessed as eight groups: four from Squamous Cell Carcinoma (SqCC; *n =* 3/3/6/5) patients and four from Adenocarcinoma (AdCa; *n =* 6/6/6/7) patients. One microarray was run per group, and groups of the same histological type were treated as biological replicates.

After protein microarray results were obtained, raw GPR files were converted to a raw excel file which contained the signal intensity for 23,059 proteins (>21,000 of which were unique, with 2000 technical replicates) for each of the sample pools. To help mitigate any batch effect variation, data was normalized. A total of six different normalization methodologies were utilized to ensure robust results. These included Cyclic-Loess, Log2, trimmed mean of m-values (TMM), and Quantile, and Robust Linear Model (RLM) processed through a widget developed for analyzing protein microarray data, called PAWER [13,14]. 

For each of the normalization methods, differential analyses using empirical Bayesian statistics (implemented via the limma package of Bioconductor), were performed comparing each actionable group (AdCa, SqCC, or combined AdCa and SqCC treated as biological replicates) versus the non-actionable high-risk screening [14]. Adjusted *p*-values were calculated using the Benjamini-Hochberg method.

Of the 23,059 autoantibodies compared, a total of 501 markers had *p*-values < 0.05, based on the moderated t-tests run comparing the actionable cohorts to the non-actionable cohorts. From these 501 markers, we identified 8 promising candidate biomarkers for discerning non-actionable against actionable nodules (either as entire category or an individual histological sub-type).

### 2.3. Custom Luminex Immunobead Assay Development (Development of Assays Used in Figure 1 Step 2 (a))

To maximize the coverage of this study and complement the 8 biomarkers discovered via the microarray studies described, an additional 18 biomarkers were assessed via Luminex assays. Of the 18, 12 were discovered via protein microarrays for a malignancy recurrence question and the other 6 were from a panel our laboratory had published, based on two-dimensional western blots and tandem mass-spectrometry [15]. This resulted in a total of 26 biomarkers of interest for further assessment via targeted Luminex assays. A custom Luminex immunobead assay was built for each of the 26 selected targets using methods we previously reported [16,17,18]. Briefly, assay construction was accomplchemistryconjugation of each recombinant protein (antigen) on a unique MagPlex bead region via standard sulfo-NHS/EDC chemistries. Conjugation efficiencies and (analytical) characteristics for each assay were evaluated against a 7-point standard curve of the corresponding anti-target antibody (rabbit polyclonal; see Table A1 for details) and read with a PE-conj, goat anti-IgG. Assay characteristics determinations included ‘working range’ assessments (limits of detection and quantitation), optimal sample dilution determinations, and assessments of performance characteristics (sensitivity, specificity, etc.). Assays were then tested for their ability to be combined into multiplex panels, using ‘leave-one-out’ testing to identify cross-reactivity issues, as we previously described [15,16]. From these efforts, 14 single-plex or multiplex panels were qualified for specimen testing that were optimized for sample dilution, primary incubation times, and secondary incubation times. 

### 2.4. Cohort Testing (Figure 1 Step 2 (a) Testing of a Large Cohort)

Custom Luminex assays developed in the previous section for the 26 candidate biomarkers were then used to assess serum from our Biomarker Development cohort (all samples in Training, Validation 1, and Validation 2). The Biomarker Development cohort consisted of 841 cases that were either screened by our Diagnostic Radiology Department for lung cancer or received an anatomic resection by our Department of Cardiothoracic Surgery. Prior to processing, subaliquots were made for each sample to ensure samples did not undergo more than two freeze–thaws. All samples were processed on 384-well plates with duplicate sampling and had a 7-point standard curve on each plate, as we previously described [17]. For overnight primary incubation, samples were diluted 1:25 in assay buffer (1X PBS/1% BSA/0.01% Tween), and 12.5 μL of diluted sample were added to the wells with 12.5 μL of MagPlex beads utilizing a ‘semi-automated’ workflow with an Agilent Bravo liquid handler. Detection of patient autoantibodies was accomplished using PE-conj., rabbit anti human IgG antibodies (obtained from Fisher Scientific manufactured by Southern Biotech). Each plate was read on a FlexMap 3D (Luminex Corp., Austin, TX, USA) to obtain median fluorescence intensity (MFI) values in xPonent v4.3 (Luminex Corp.). The concentrations of each marker for each sample in the large cohort was calculated using Belysa v1.1 Software. The software mapped the MFI signal from the sample well to the 4PL logistic curve produced based on the 7-point standard curve of the anti-target antibody. Replicates which had a coefficient of variation equal to or greater than 50% were removed, as were reads which had <30 beads/well, based upon thresholds recommended by Luminex and others [19]. 

### 2.5. Luminex Data Pre-Processing and Analysis (Figure 1 Step 2b) Assessment of Targets Individual Performance for Discerning Actionable from Non-Actionable Samples)

Boxplots comparing actionable nodule patients to non-actionable nodule patients were produced for all biomarkers tested via Luminex assays, with *p*-values determined via Mann–Whitney (two-sided) U [20,21,22]. Outliers were removed for the creation of the boxplots and for the determination of the individual marker *p*-values. ROC/AUC curves were produced for each of the individual biomarkers utilizing the pROC package in R [23]. The top 10 most significant biomarkers were selected based on the Mann–Whitney U test results. For these biomarkers, a generalized linear model was trained on 70% of the data, with an optimal cut-off selected that produced the highest true positive rate. The developed models were then applied to the remaining 30% of the cohort, and performance characteristics were assessed. 

### 2.6. Development of a Multianalyte Panel for Patient Risk Stratification (Figure 1 Step 3)

Prior to any machine learning, data from the Biomarker Development cohort was split randomly into three separate sets: Training (*n =* 565), Validation 1 (*n =* 93), and Validation 2 (*n =* 183; aka a ‘test’ cohort). Biomarkers with at least 80% non-missing values were considered for panel development. 

For the purposes of variable selection, each possible 6 or 7 marker combination of 19 biomarkers was considered. A random forest prediction model was developed for each of the combinations using the “Training” cohort [15,24]. Prediction performance of each of the random forest models developed were determined based on the OOB error of the model in the training set and the prediction accuracy of the model in the Validation 1 cohort. The marker combination that resulted in a random forest model with the best performance metrics was selected as the final panel.

The final random forest model was trained using the Training set, based on the 6-marker panel selected. The performance characteristics of this model at classifying actionable from non-actionable nodules was calculated for both the Validation 1 and Validation 2 cohort. 

An optimal cut-off was created to minimize ‘false-negative’ results given that sensitivity was considered paramount to our application. The actionable “vote” score cut-off was the proportion of decision trees in the model which assessed the case as actionable; their predictive performances in the Validation 1 cohort were used to create an ROC curve [24]. An optimal threshold was determined by detecting a “vote” score cut-off which offered >95% sensitivity in the Validation 1 cohort, while minimizing the amount of ‘false positives’. This cutoff value was then used to recalculate panel performance characteristics against the Validation 2 cohort [25]. This process is defined in Figure 1 Step 3c. Finally, the performance metrics were further evaluated in the clinically distinct groups for each of the different cohorts tested. 

## 3. Results

### 3.1. Patient Population for the HuProt^TM^ Microarrays for the Discovery of Novel Lung Cancer Early Detection Targets

A total of 62 samples were included in the Discovery cohort used for the protein microarray study, with patient clinical and demographic information provided (Table 1). These samples were combined into 10 sample pools (8 ‘actionable’ and 2 ‘non-actionable’) that were utilized to probe the microarrays. 

To diversify the coverage of the biomarkers selected, a range of common lung pathologies and malignancies relevant to lung cancer screening were included in this cohort, given the potential these would have unique molecular profiles. Within the lung malignancy cases, all samples included had actionable nodules based on Lung-RADS v1.1 and Lung-RADS v2022. Additionally, all malignancy cases were confined to T_1–3_N_0_M_0_. All patients with non-actionable nodules qualified for lung cancer screening based on current USPSTF guidelines (i.e., were between the ages of 50–80, had at least a 20 pack–year smoking history, and were current smokers or had quit within the last 15 years) at the time of sample collection. A single sample possessing a non-malignant nodule was inadvertently included in one of the ‘non-actionable’ groups. 

### 3.2. Autoantibodies with Differential Signal in Patients with ‘Actionable’ vs. ‘Non-Actionable’ Nodules via HuProt™ Protein Microarrays

**Figure 2:**
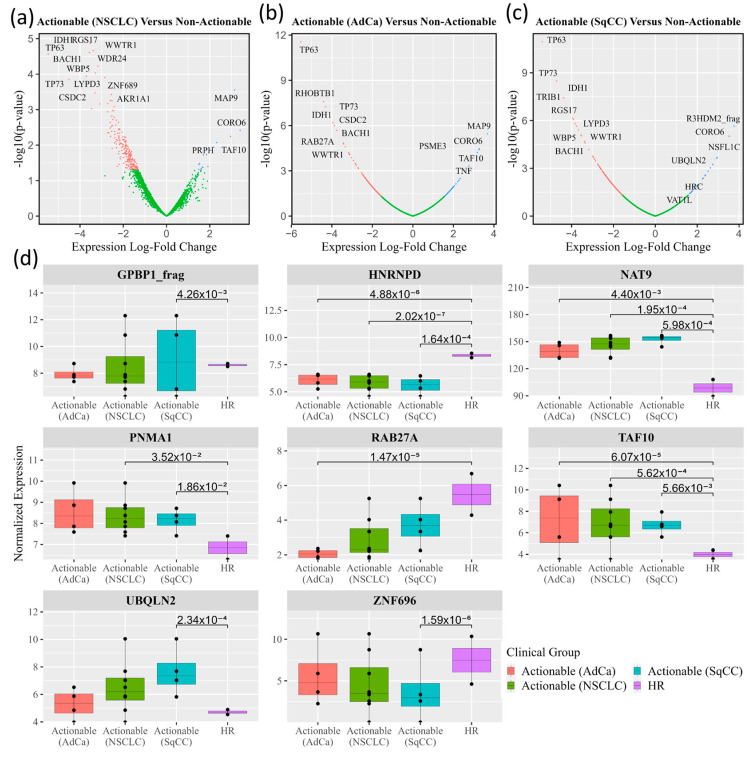
Candidate Biomarker Performance for Actionable Versus Non-actionable Nodules within the HuProt Microarray. Novel biomarkers for discerning actionable versus non-actionable nodules were determined by comparing autoantibody signal levels between microarrays utilizing empirical Bayesian statistics with the Bioconductor package in R. Adjusted *p*-values were determined utilizing the Benjamini–Hochberg method. Volcano plots were created for comparisons made between high-risk screening patients with non-actionable nodule sample pools and different actionable nodule subsets, those with (**a**) NSCLC (both AdCa and SqCC) (**b**) AdCa, and (**c**) SqCC. Autoantibodies with differential signals that had an absolute value Log Fold Change (LFC) > 0.6 and *p*-values < 0.05 are in red if elevated in the non-actionable group, and in green if they are elevated in the actionable group. Biomarkers which are labeled had *p*-values < 0.01 and absolute value log-fold change > 2. (**d**) Specific biomarkers selected for further analysis are displayed as boxplots.

Normalized data from the HuProt™ protein microarrays were processed using empirical Bayesian statistics and displayed with volcano plots to contrast autoantibodies that differentially associate with ‘actionable’ or ‘non-actionable’ nodules, as shown in Figure 2a. Further delineation of the ‘actionable’ group based on histology provided similar plots for AdCa (Figure 2b) and SqCC (Figure 2c) relative to the ‘non-actionable’ group. A total of 501 markers were found to be differentially recognized (*p* < 0.05) in the actionable sample pools (AdCa, SqCC, or AdCa and SqCC) compared to non-actionable sample pools. 

Candidate biomarkers were preferentially selected for further development if the marker was (1) relevant to more than one of the comparisons of interest (i.e., AdCa versus high-risk and SqCC versus high-risk), (2) significant after a Benjamini–Hochberg correction (i.e., adjusted *p*-value), (3) significant in more than one normalization method, (4) if they were elevated in the actionable group compared to the non-actionable group. 

Based on these criteria, eight candidate biomarkers (GPBP1, HNRNPD, NAT9, PNMA1, RAB27A, TAF10, Ubiquillin 2, ZNF696) were selected for development based on ability to distinguish ‘actionable’ from ‘non-actionable’ nodules, with ‘box and whisker’ plots (or boxplots) for these biomarkers shown in Figure 2d.

To maximize the study’s coverage of biomarkers, 18 additional biomarkers were assessed, which our laboratory found had relevancy to early detection of lung cancer but were not discovered for the express purpose of discerning actionable versus non-actionable nodules. Utilizing Luminex assays, we tested a total of 26 different biomarkers within the patient cohort. These biomarkers included the 8 candidate biomarkers discovered via the microarray for discerning actionable versus non-actionable cohorts, 12 candidate biomarkers with relevance to lung cancer screening questions described in methods, and 6 biomarkers that our laboratory had previously published [15]. Biomarkers were tested with a total of 14 different multiplexes/single plexes, as shown in Table 2.

### 3.3. Charactersitics of the Biomarker Development Cohort with Subgroups for the Classification Model Development and Assessment Provided

Custom Luminex assays were used to assess levels of each biomarker within the Biomarker Development cohort (*n =* 841). Of the 841 patients within the cohort, 449 patients had actionable nodules and 392 patients had non-actionable nodules. Pathological (histological) diagnostic classifications of the cohort include patients grouped as histologically benign nodules (BN) (*n =* 101), early-stage NSCLC (*n =* 245), high-risk screening (*n =* 466), and patients with other malignancies (*n =* 29) who qualify for screening based on current USPSTF guidelines. Within the lung malignancy samples, a total 265 samples were tested (including small-cell lung cancer and carcinoid cases), 162 samples were T_1a-b_N_0_M_0_, 52 were T_2a-b_N_0_M_0_, 29 were T_3_N_0_M_0_, and 11 were T_4_N_0_M_0_. Patient demographic information and pathological (histological) diagnostic information for this cohort are provided in Table 3. Further breakdown of the nodule development, metastasis, and histological grouping can be seen in Table A2. 

**Figure 3:**
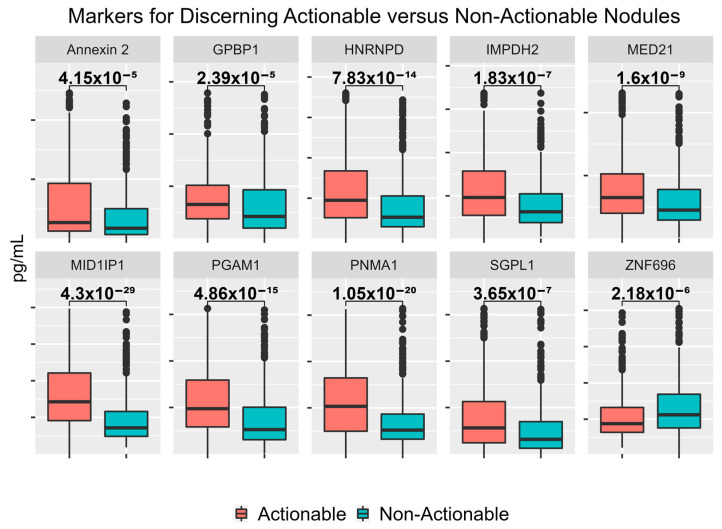
Boxplots for the Highest Performing Biomarkers for Discerning Between the Actionable and Non-actionable Cohorts. The top 10 most significant biomarkers of the 26 biomarkers assessed within the large cohort are shown as ‘box-and-whisker’ plots (or boxplots).

Of the 26 candidate biomarkers tested, 17 were significant for discerning actionable cases from non-actionable cases, with *p*-values < 0.05. Looking specifically at the performance of the eight novel markers discovered utilizing the HuProt microarray (GPBP1, HNRNPD, NAT9, PNMA1, RAB27A, TAF10, Ubiquillin 2, ZNF696) for discerning actionable versus non-actionable nodules, seven were significant with *p*-values < 0.05. Performance for all 26 biomarkers tested are listed in Table 4, whereas boxplots for the top 10 most significant markers are shown in Figure 3.

### 3.4. Performance of Logistic Regression Produced from Top Biomarkers

**Figure 4:**
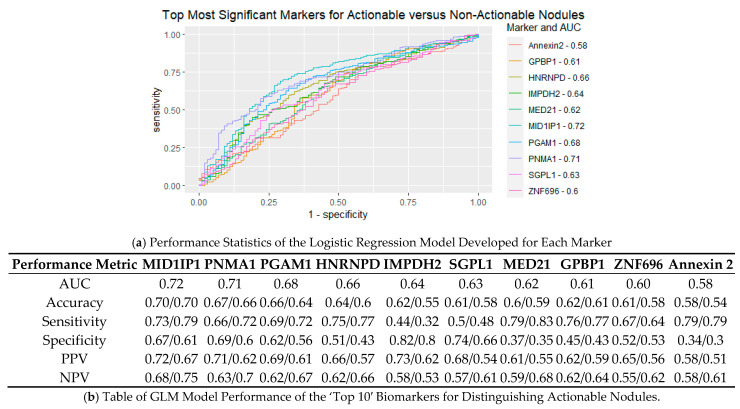
Assessment of Logistic Regression Models for Top Performing Biomarkers. The performance characteristics of general logistic regression models developed for each of the top 10 most significant biomarkers are provided. Panel (**a**) illustrates a ROC curve for each of the biomarkers based on a training cohort. Area under the curve (AUC) values can be seen next to the marker name in the legend. Panel (**b**) details the performance metrics for each of the general logistic models are shown for the training set used to create an optimal cut-off value and an independent test set.

After determining the significance of each marker, we wanted to ascertain each marker’s individual ability to discern between actionable and non-actionable cases based on generalized linear models. Logistic regression models were trained for each of the top 10 most significant biomarkers based on 60% of the total collected data, with 40% of the data left for a testing set. ROC curves created for the training set are illustrated in Figure 4a, and calculated performance metrics listed in Figure 4b. Performance metrics were determined for the training and testing sets of the generalized linear models based on the optimal cut-off determined from the training set. AUCs for the top biomarkers ranged from 0.58–0.72, with MID1IP1 having the highest AUC (0.72). Interestingly, while MID1IP1 had the highest AUC, MED21 had the highest sensitivity, with 79% sensitivity in the training cohort and 83% sensitivity in the testing. While accuracy is an important metric for determining a model’s performance, due to the nature of the test we are developing, high sensitivities are of higher importance as false negatives result in delayed diagnosis of cancer, whereas a false positive leads to test follow-up through LDCT. 

To further assess the performance of the individual biomarkers, we broke down the performance by clinically distinct cohorts: benign cohort, malignant, or high-risk screening cohort. Breakdowns by total accuracy in classification can be seen in Table A3 for the top five most significant biomarkers. For lung malignancy, the accuracy of the models based on the individual biomarkers ranged from 50–82%, with the best marker being HNRNPD. For histologically benign cases, the accuracy was 44–82%, with HNRNPD and MID1IP1 both having an actionable classification accuracy of 82%. Finally, for the screening cohort, the classification of samples into actionable or non-actionable subsets was 45–68%, with the highest accuracy within the IMPDH2 biomarkers.

### 3.5. Creation of Random Forest Model for Determining Actionable versus Non-Actionable Nodules

After determining the performance of the individual biomarkers, we aimed to develop a panel of biomarkers to obtain optimized performance characteristics for identifying patients with actionable versus non-actionable nodules. For this, our Biomarker Development Cohort was subdivided into Training, Validation 1, and Validation 2 sub-cohorts, as defined in Section 2.5 and with characteristics shown in Table A4.

Machine learning models have been shown to help improve overall performance of biomarker panels and have become standard for the purpose of developing clinical biomarker tests. Our laboratory had previously published a panel in which we utilized a random forest machine learning model to develop a model with good sensitivity and specificity for discerning between four clinically distinct groups: early-stage lung cancer, osteoarthritic, non-neoplastic nodules, and COPD/asthma patients [15]. Based on this, we decided to attempt to develop a random forest model for discerning between actionable and non-actionable nodules. 

### 3.6. Development of an Preliminary Biomarker Panel via Machine Learning

The objective of this step was to determine the optimal combination of biomarkers for risk-stratifying patients for potential identification of an actionable nodule via LDCT-based screening protocols. For feature selection, we calculated performance for every unique combination of 6 and 7 biomarkers of 19 biomarkers identified from our microarray and from our previously published panel. A total of 19 biomarkers were utilized as 7 of the 26 biomarkers were eliminated from this analysis (DR1, IMPDH2, NAT9, IKZF5, KEAP1, RAB27A, Ubiquillin 2) due to >20% missing data-based on percent coefficient of variation and low bead counts. For the rest of the biomarkers, imputation was used within the training cohort to maximize the number of samples that could be used for model development. We examined each combination of six and seven biomarkers, training a random forest model and recording its performance metrics seen within the Training and Validation 1 cohort. The exploration with all possible six marker combinations tested a total of 27,132 different combinations, and the one with all possible seven marker combinations tested a total of 50,388 different combinations. The top outputs of each 6- and 7-marker combination were compared, and, given that the accuracy was less than 2% different between the models, we opted to utilize the 6-marker model to economize future studies with this panel. 

The panel which showed the greatest accuracy within the Validation 1 cohort was Annexin 2, DCD, MID1IP1, PNMA1, TAF10, and ZNF696. Three of these biomarkers (PNMA1, TAF10, and ZNF696) were chosen based on the HuProt microarray discovery outlined in Section 3.1 and Section 3.2. Annexin 2 was part of a biomarker panel our laboratory had previously published, with DCD and MID1IP1 both holding value for early detection based on previous laboratory studies. Of the biomarkers chosen, four (MIDIP1, PNMA1, Annexin 2, and ZNF696) had individual *p*-values < 0.01, one (TAF10) had a *p*-value < 0.05, and, interestingly, one (DCD) was not significant (*p* > 0.05) for discerning actionable from non-actionable cases in the Biomarker Development cohort. We further tested the panel on the Validation 2 cohort which was not utilized for panel determination or optimization purposes. The Validation 2 cohort consisted of 183 patients, with 84 patients at high-risk of lung cancer with non-actionable nodules and 99 patients with actionable nodules (solid pulmonary nodules > 6 mm). The accuracy within the third cohort was 72.48%, with a sensitivity of 76.62% and specificity of 68.06%. 

### 3.7. Performance of Final Optimized Panel for Patient Risk Startification

Given that the objective of this study was to develop a risk stratification tool to pre-screen individuals for LDCT-based lung cancer screening protocols, we aimed to focus on minimizing ‘false negative’ findings to reduce the potential of missing a malignancy at the cost of some ‘false positives’. To this end, we created an ROC curve on the Validation 1 cohort. Assessing different cut-points used for the ROC curve, we selected the cut-off which had >95% sensitivity within the Validation 1 cohort with the least cost to specificity. The resulting risk cut-off was 0.334 as opposed to the standard 0.5, for which the model was originally trained.

**Figure 5:**
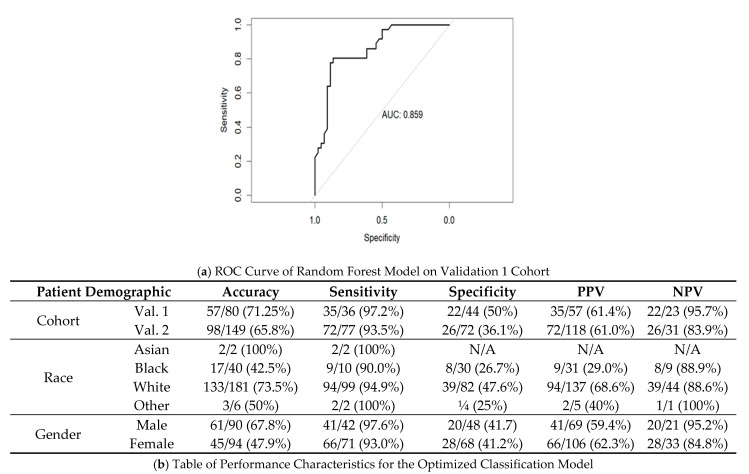
Assessment of Sensitivity Optimized Classification Model. (**a**) The ROC developed from predicted risks determined by the random forest model in the Validation 1 cohort. From these predicted risk values, thresholds (or ‘cut-offs’) were chosen, which allowed for the highest specificity with a sensitivity of 95%. (**b**) The model performance when utilizing the optimized cut-off value in Validation 1 and Validation 2 cohorts, with further resolution by race and gender.

The performance characteristics were recalculated within the Validation 1 and Validation 2 cohort, with the resulting ROC curve illustrated in Figure 5a. Within the Validation 1 and Validation 2 cohorts, sensitivity was high, at 97.2% and 93.5%, respectively. With the increase in sensitivity there was a decrease in specificity to 50% and 36.1%, respectively. Figure 5b depicts the accuracy of the model against different racial and gender breakdown. Notably, negative predictive values (NPVs) between white and African-American subgroups were comparable. 

The panel was further assessed for its performance for different clinically distinct subgroups. The Validation 1 and Validation 2 cohorts were further subdivided into three main groups: malignancy cases, histologically benign cases, and high-risk screening cases. These subgroups were then evaluated based on histological diagnosis and nodule presentation. The accuracy of the classification of each of these subgroups is shown in Table 5. 

For the malignancy cases (*n =* 71), there was an overall classification accuracy of 94.4%, with all malignancy cases designated as actionable nodules (i.e., >6 mm). Performance was high across all subsets of lung malignancy, with ranges from 90% to 100% accuracy. In the future, it may be of interest to assess additional biomarkers which hold value for discerning SqCC to help supplement the current panel and improve performance within this subset of patients. 

Histologically benign cases are made up of serum samples obtained from patients who underwent lung resection and were determined by pathology to have “benign” nodules. Within this cohort, there was a high accuracy of 83.3%. Notably, within the benign cases, the non-actionable cases were the only cases that were misclassified. This suggests that this panel may perform very well at identifying patients with a benign lung disease.

Finally, we assessed the performance in the high-risk screening cohort. The high-risk screening cases made up the majority of the non-actionable cases assessed in this study. Of the screening cohort, 51.1% of the cohort was properly classified as having actionable or non-actionable nodules. The performance within the screening cohort with actionable nodules was high, with 21/23 samples within Validation 1 and Validation 2 properly classified. Within the screening cohort with non-actionable nodules, performance was worse, with only 48/112 cases being identified as non-actionable. This is likely a result of the panel being optimized for the identification of actionable nodules, with false positives having less importance due to the availability of other testing mechanisms. Additionally, for 56 of the 64 misclassified non-actionable cases, subsequent LDCT screening results were available and assessed. During subsequent screens, 6/56 had actionable nodules, with three being classified as 4A, two being classified as 4B, and one classified as 3 via Lung-RADS v1.1 or v1.0, depending on date of screening. Taking these cases into account, this would potentially increase the specificity of this test to 54.5% in the Validation 1 cohort and 41.7% in the Validation 2 cohort.

## 4. Discussion

NSCLC is the leading cause of cancer related mortality world-wide, much of which can be attributed to late diagnosis. Screening is key to detecting malignancies early, and the current mechanism for screening is annual LDCTs within high-risk populations. While LDCT can be extremely sensitive for the detection of lung nodules, with estimates reported from 59% to 100%, specificity of LDCT tends to be lower, with estimates which can vary dramatically from 26.4% to 99.7% [26]. These detection ranges highlight the variability with this screening method, as radiologists’ training may dramatically impact test performance. Improved methods for identifying patients of interest for LDCT screening, based on circulating biomarkers, might help enhance the specificity of the overall lung cancer screening paradigm. To this end, we developed a blood-based biomarker test which could aid in the identification of patients with actionable nodules. It is estimated that only about 10–20% of high-risk patients will have actionable nodules [9,27], so identifying these patients using this test might avoid many unnecessary (or ‘futile’) LDCTs and reduce ‘false-positive’ rates. A simple blood-based test would be much more accessible than LDCT, thus increasing the convenience of the initial evaluation (a ‘pre-screen) and potentially increasing uptake rates, which are currently low (6–14%) among patients [5,26]. We have developed a blood-based test which has a sensitivity of 93.5%, with a specificity of 36.1%. According to multiple studies, approximately 80–90% of patients who qualify for screening do not harbor actionable nodules [5,8,27]. This means that our test could decrease the number of futile scans by approximately 30% while maintaining the sensitivity levels observed in LDCT. 

Our efforts to identify markers that held value for discerning patients with actionable versus non-actionable nodules were focused on the discovery of circulating autoantibodies. To avoid autotoxicity, B-cells generally have a central and peripheral tolerance, which prevents them from producing autoantibodies targeted at self-antigen [28]. However, it is believed that B-cells can overcome their peripheral tolerance when tumors produce autoantigens that are overly expressed, expressed in areas of the body they typically are not, or are a result of a mutation that leads to an antigenic (or neoantigenic) protein [29]. Thus, studies have noted the presence of autoantibodies within cancer and at very early stages of tumor development, making them ideal screening biomarker candidates [29].

Multiple papers have focused on the development of blood-based autoantibody biomarker tests for lung malignancy detection [30]. Our laboratory previously published an autoantibody panel which consisted of Annexin 1, Annexin 2, IMPDH2, HSP70, PGAM1, and Ubiquillin 1, and properly classified 93% within five clinically distinct groups: osteoarthritis, “cancer-free” control, asthma/COPD, benign nodule, and NSCLC [16]. These markers were included within this study. Other laboratories have also studied autoantibodies for lung cancer. Two studies by Huang et al. (2020) and Jia et al. (2014) assessed autoantibodies used in tandem with LDCT nodule size results to create a lung malignancy predictive model with sensitivities of 70.1% and 80% and specificities of 72.6% and 89% respectively [31,32]. One of the most extensively studied examples of an autoantibody lung cancer biomarker tests is Early-CDT. Early-CDT has consistently high specificity (80–90%), however, its sensitivity is lower at approximately 33–39% [33,34]. While all of these tests hold merit for complimenting LDCT, none have value as a pre-screening tool as they lack the sensitivity required for a pre-screening test. To our knowledge, this study is the first to describe a method capable of impacting patient selection criteria for LDCT based on a panel of biomarkers trained to identify patients with actionable nodules according to Lung-RADS criteria. 

One limitation of our study is that, at the time of model training, Lung-RADS report information was not available for nodules between 4 mm and 6 mm, so it could not be determined if they would be actionable. Thus, four cases were misclassified as non-actionable within our Training cohort. This misclassification may have resulted in slightly worse performance in the Validation 1 and Validation 2 cohorts; however, since these samples only made up 4/565 samples, this is unlikely. Another study limitation that may have led to misclassifications is the limited availability of patient follow-up information for those without a definitive pathological diagnosis (i.e., the high-risk screening cohort). To minimize this source of error, all available follow-up reports in our electronic medical record system were assessed, which totaled 459 out of the 466 high-risk screening patients. Within the high-risk screening patient group, seven patients did not return to Rush University after their initial appointment; thus, no follow-up was available. Follow-up ≥1 year was available for 456/466 patients, and only two patients went on to develop lung origin malignancy post-blood draw. One of these patients had lung malignancy 2 years after blood draw, with another 4 years after. Three patients had their latest reported follow-up <1 year after sample collection but were cancer free at that point. Since reports were found for most high-risk screening patients, and no documented lung malignancy within a year of blood draw was recorded, we believe this was unlikely to affect the outcomes of our study.

## 5. Conclusions

With the completion of this study, we have identified biomarkers for the purposes of discerning actionable versus non-actionable nodules, utilizing protein microarrays. We then determined individual biomarker performance metrics for discerning between actionable and non-actionable cohorts based on general linear models. Finally, we developed a machine learning model based on circulating levels of six biomarkers (Annexin 2, MID1IP1, PNMA1. TAF10, and DCD) that have high sensitivity (97.2% in Validation 1 and 93.5% in Validation 2) for the purposes of identifying patients with actionable nodules. 

Within the eligible screening population, only about 10–20% have actionable pulmonary nodules. Patients with actionable nodules have a risk 9–96 times higher for developing lung cancer compared to the rest of the high-risk screening cohorts. Those at high-risk without actionable nodules are estimated to have a similar lung cancer rate, 0.1%, as never-smokers; estimates are 0.1% in females and 0.2% in males [9,35]. This suggests that our biomarker test could identify a small population (10–20%) within the general screening population who would benefit from LDCT. The majority of the screening patients may only need the annual biomarker blood test, which is simple, cost-efficient, and more easily accessible.

This panel of biomarkers may help improve the current initial lung cancer screening paradigm. In future, more validation studies with a larger patient cohort consisting of high-risk screening patients will be of interest for model optimization on large populations with more reflective population breakdowns.

## 6. Patents 

The method described in this manuscript is protected under US Provisional Application Number 63/453,250 filed on 20 March 2023.

## Figures and Tables

**Table 1:** Clinical characteristics of the Discovery Cohorts used for the HuProt™ protein microarray studies. Basic clinical characteristics of all samples comprising the Discovery Cohort are provided as used for the HuProt microarray studies. The adenocarcinoma (AdCa) and squamous cell carcinoma (SqCC) samples are each divided across four sample pools, whereas the non-actionable samples are divided across two sample pools.

Group		Actionable		Non-Actionable
		AdCa	SqCC	
		(*n =* 25)	(*n =* 17)	(*n =* 20)
Age (years)	Median (Range)	70 (56–83)	75 (60–79)	65 (58–71)
Gender	Male (%)	13 (50%)	8 (47.1%)	10 (52.6%)
Lesion Size (mm)	Median (range)	24.8 (11–38)	28 (11–35)	3 (2–10)
AJCC Stage	IA1	1	N/A	
	IA2	8	6	
	IA3	4	7	
	IB	12	3	
	IIB	N/A	1	

**Table 2:** Candidate biomarkers tested with panel composition indicated. In Table 2 each target for which a Luminex assay was developed and used to test the Biomarker Development cohort is listed out. The targets are organized by their multiplex/single plex group. Multiplexed markers can be measured in tandem within patient serum.

Single-Plex Assay	Multiplex Assay
Annexin 2	Annexin 1 and NIP30
KEAP1	CFAP36, MID1IP1
HNRNPD	DCD, MED21, TAF10, ZNF696
IMPDH2	Dr1, HSP70
NAP1L5	GPBP1, MYBPH, PGAM1
Ubiquillin 1	IKZF5, NAT9, PNMA1
Ubiquilllin 2	RAB27A, SGPL1, TP53

**Table 3:** Biomarker Development Cohort. In Table 3, the cohort that was tested for the 26 potential biomarkers via Luminex assays is broken down by patient demographics.

Patient Demographic	Total	Non-Actionable	Actionable
	*n =* 841	*n =* 392	*n =* 449
Gender			
Male (%)	353 (41.97%)	161 (41.07%)	192 (42.76%)
Age, years Median	67	65	69
Minimum	41	47	41
Maximum	88	82	88
Diagnosis			
NSCLC	245	1	244
AdCa		1	160
SqCC		0	73
AdCa/SqCC Mixed		0	9
NSCLC (Not Specified)		0	2
Malignancy, non-NSCLC	29	1	28
Carcinoid (G1/G2)		0	8
Large-Cell/SCLC (G3)		0	12
Metastasis (Not Lung Cancer)		1	8
Benign	101	11	90
Granuloma		2	27
Hamartoma		0	14
Fibrosis/Scar/Inflammation		9	37
Infection/Org. Pneumonia		0	5
Other		0	7
Not Assessed *	466	379	87
Stable or Resolving		123	31
Interval Increase in Size		2	4
New Nodule/Unknown Growth		202	50
Mix of New/Stable Nodules		1	0
No Nodule/Non-Specified		51	2

* Patient did not undergo surgery, and thus, results are based on radiological presentation.

**Table 4:** Performance of 26 Biomarkers for Discerning Between the Actionable and Non-actionable Cohorts. Individual biomarker performances were compared between actionable and non-actionable nodules via the Mann–Whitney U Test.

Protein Name	Uniprot ID	Non-Actionable Median (Range), ng/mL	Actionable Median (Range), ng/mL	*p*-Value
MID1IP1	Q9NPA3	71.25 (17.85–388.06)	142.49 (0.14–397.30)	4.30 × 10^−29^
PNMA1	Q8ND90	15.59 (0.19–94.31)	30.99 (0.25–93.93)	1.05 × 10^−20^
PGAM1	P18669	5.25 (0.11–30.96)	9.74 (0.02–31.27)	4.86 × 10^−15^
HNRNPD	Q14103-1	13.23 (0.19–85.85)	23.71 (0.09–90.12)	7.83 × 10^−14^
MED21	Q13503	45.12 (1.59–229.99)	65.03 (0.1–231.13)	1.60 × 10^−9^
IMPDH2	P12268	316.41 (30.23–1685.81)	481.49 (8.89–1686.36)	1.83 × 10^−7^
SGPL1	O95470	7866.32 (6.02–77,865.4)	14,005.25 (0.15–78,349.22)	3.65 × 10^−7^
ZNF696	Q9H7X3	112.12 (4.38–404.82)	87.71 (21.5–393.02)	2.18 × 10^−6^
GPBP1	Q86WP2	42.09 (0.18–275.87)	65.18 (0.05–278.53)	2.39 × 10^−5^
Annexin 2	P07355	17.27 (0.01–228.71)	26.81 (0.28–245.76)	4.15 × 10^−5^
NAT9	Q9BTE0	1155.48 (45.72–4082.57)	1551.26 (7.44–4304.02)	8.67 × 10^−5^
TP53	P04637	43.78 (0.75–234.93)	59.04 (0–236.63)	0.0003
Annexin 1	P04083	0.96 (0.01–8.44)	1.34 (0–8.51)	0.014
NIP30	Q9GZU8	4.91 (0.03–29.18)	5.96 (0.01–29.71)	0.014
TAF10	Q12962	164.68 (5.48–584.71)	147.65 (26.31–567.49)	0.023
RAB27A	P51159	0.54 (0.01–2.70)	0.67 (0.01–2.73)	0.027
KEAP1	Q14145	6.12 (0.06–29.3)	8.24 (0.01–30.98)	0.041
Ubiquillin 1	Q9UMX0	4.73 (0.2–42.17)	484.48 (9.02–5403.48)	0.077
HSP70	P0DMV9	0.7 (0–2.73)	0.8 (0.09–2.74)	0.163
MYBPH	Q13203	436.92 (3.36–1721.33)	385.88 (0.66–1760.32)	0.211
NAP1L5	Q96NT1	587.41 (2.02–2535.53)	632.27 (0.51–2399.45)	0.234
IKZF5	Q9H5V7	46.96 (0.06–331.96)	49.68 (0.07–338.83)	0.258
Ubiquillin2	Q9UHD9	99.87 (2.39–499.05)	84.37 (0.65–506.10)	0.454
Dr1	Q01658	18.05 (0.01–97.89)	20.58 (0.02–94.76)	0.507
DCD	P81605	2022.34 (71.6–6775.07)	2120.54 (345.25–6768.67)	0.539
CFAP36	Q96G28	27.08 (0.04–109.11)	27.74 (0.03–112.46)	0.631

**Table 5:** Performance of Sensitivity Optimized Model broken Down by Clinically Distinct Cohort. Performance characteristics of the sensitivity optimized biomarker panel are broken down by clinically distinct groups.

Subgroups	Overall Accuracy	Accuracy (Actionable)		Accuracy (Non-Actionable)	
	Combined	Validation 1	Validation 2	Validation 1	Validation 2
Metastasis	1/1 (100%)	NA	1/1 (100%)	NA	NA
AdCa	42/44 (95.5%)	15/16 (93.75%)	27/28 (96.43%)	NA	NA
AdCa/SqCC Mixed	1/1 (100%)	1/1 (100%)	NA	NA	NA
SqCC	18/20 (90.0%)	6/6 (100%)	12/14 (85.7%)	NA	NA
NSCLC (General)	1/1 (100%)	NA	NA|1/1 (100%)	NA	NA
Carcinoid	2/2 (100%)	1/1 (100%)	1/1 (100%)	NA	NA
Small-Cell	2/2 (100%)	1/1 (100%)	1/1 (100%)	NA	NA
Malignancy Totals	67/71 (94.4%)	24/25 (96.0%)	43/46 (93.5%)	NA	NA
Granuloma	3/3 (100%)	1/1 (100%)	2/2 (100%)	NA	NA
Hamartoma	2/2 (100%)	NA	2/2 (100%)	NA	NA
Fibrosis/Scarring/Inflammation	12/16 (75%)	2/2 (100%)	10/10 (100%)	0/2 (0%)	|0/2 (0%)
Infection/Pneumonia	1/1 (100%)	NA	1/1 (100%)	NA	NA
Other Non-Malig. Nodule	2/2 (100%)	1/1 (100%)	1/1 (100%)	NA	NA
Benign Totals	20/24 (83.3%)	4/4 (100%)	16/16 (100%)	0/2 (0%)	0/2 (0%)
Stable or Resolving Nodule	24/57 (42.1%)	5/5 (100%)	6/7 (85.7%)	6/16 (37.5%)	7/29 (24.1%)
Interval Increase in Size	0/1 (0%)	NA|0/1 (0%)	0/1 (0%)	NA	NA
New Nodule/Unknown Growth	37/61 (60.7%)	3/3 (100%)	7/7 (100%)	11/19 (57.9%)	16/32 (50%)
No Noted Nodule	8/16 (50%)	NA	NA	5/7 (71.4%)	3/9 (33.3%)
Control Totals	69/135 (51.1%)	8/8 (100%)	13/15 (86.7%)	22/42 (52.4%)	26/70 (37.1%)

## Data Availability

The data presented in this study will be openly available in Mendeley Data at https://doi.org/10.17632/d2p7zc54gk.1 upon manuscript publication.

## Appendix A

**Table A1:** Table of Immunoreagents used in Biomarker Development.

Marker	Uniprot	Single-Plex/Multiplex	Antibody Catalog	Source
Annexin 1	P04083	1	21990-1-AP	Proteintech
NIP30	Q9GZU8	1	LS-C667168	Lifespan Biosciences
Annexin 2	P07355	2	11256-1-AP	Proteintech
CFAP36	Q96G28	3	LS-C664461	Lifespan Biosciences
MID1IP1	Q9NPA3	3	LS-C80861	Lifespan Biosciences
DCD	P81605	4	LS-C754340	Lifespan Biosciences
MED21	Q13503	4	CSB-PA070304	Cusabio
TAF10	Q12962	4	H00006881-D01P	Novus Biological
ZNF696	Q9H7X3	4	LS-C101596	Lifespan Biosciences
Dr1	Q01658	5	LS-C755318	Lifespan Biosciences
HSP70	P0DMV9	5	14887-1-AP	Proteintech
KEAP1	Q14145	6	TA590238	OriGene
GPBP1	Q86WP2	7	LS-C753825	Lifespan Biosciences
MYBPH	Q13203	7	LS-C500819	Lifespan Biosciences
PGAM1	P18669	7	16126-1-AP	Proteintech
HNRNPD	Q14103-1	8	LS-C211799	Lifespan Biosciences
IKZF5	P04083	9	HPA051574	Atlas Antibodies
NAT9	Q9BTE0	9	ABIN631510	Antibodies-online
PNMA1	Q86WP2	9	H00009240-D01P	Novus Biological
IMPDH2	P04083	10	LS-C666439	Lifespan Biosciences
NAP1L5	P04083	11	LS-C680924	Lifespan Biosciences
RAB27A	Q86WP2	12	LS-C662585	Lifespan Biosciences
SGPL1	O95470	12	H00008879-D01P	Novus Biological
TP53	Q9NPA3	12	PAB12719	Abnova
Ubiquillin 1	Q9NPA3	13	23516-1-AP	Proteintech
Ubiquillin 2	Q9NPA3	14	LS-C661407	Lifespan Biosciences
IgG Goat anti-Human, R-PE, Polyclonal	RRID: AB_2795648	N/A	OB204009	Fisher Scientific
IgG Goat anti-Rabbit, R-PE, Polyclonal	P01870	N/A	OB403009	Fisher Scientific

**Table A2:** Breakdown of staging of lung malignancy cases within the Training, Validation 1 and Validation 2 Cohorts.

Stage	AdCa	AdCa/SqCC	SqCC	NSCLC	Carcinoid	Large Cell
T1a	24	1	5	1	2	4
Not Available	0	0	1	0	1	0
N0	24	1	4	0	1	4
N1	0	0	0	1	0	0
T1b	65	4	16	0	2	1
Not Available	2	0	1	0	0	0
N0	63	4	15	0	2	1
N1	0	0	0	0	0	0
T1c	16	1	14	0	2	4
Not Available	1	0	0	0	0	0
N0	15	1	14	0	2	4
N1	0	0	0	0	0	0
T2a	25	1	9	0	1	2
Not Available	0	0	0	0	0	0
N0	24	1	9	0	1	2
N1	0	0	0	0	0	0
N1, M1b	1	0	0	0	0	0
T2b	4	0	9	1	0	0
Not Available	0	0	0	0	0	0
N0	4	0	9	1	0	0
N1	0	0	0	0	0	0
T3	11	2	14	0	1	1
Not Available	1	0	1	0	0	0
N0	10	2	13	0	1	1
N1	0	0	0	0	0	0
T4	6	0	5	0	0	0
Not Available	1	0	2	0	0	0
N0	5	0	3	0	0	0
N1	0	0	0	0	0	0
Not Available	10	0	1	0	0	0

**Table A3:** Performance of GLM for the ‘Top Five’ Biomarkers.

SUBGROUP	DIAGNOSIS	IMPDH2	HNRNPD	PGAM1	PNMA1	MIDIP1
MALIGNANCY	Lung Metastasis	0.67 (4/6)	1 (8/8)	0.88 (7/8)	0.86 (6/7)	0.75 (6/8)
	AdCa	0.48 (47/97)	0.81 (114/140)	0.74 (105/142)	0.68 (92/135)	0.8 (118/148)
	Mixed AdCa/SqCC	0.6 (3/5)	0.75 (6/8)	0.71 (5/7)	0.63 (5/8)	0.67 (6/9)
	SqCC	0.57 (24/42)	0.94 (61/65)	0.88 (58/66)	0.82 (53/65)	0.85 (57/67)
	NSCLC (Unspecified)	0 (0/1)	0 (0/1)	0.5 (1/2)	0.5 (1/2)	0.5 (1/2)
	Carcinoid	0 (0/4)	0.5 (4/8)	0.43 (3/7)	0.38 (3/8)	0.5 (4/8)
	Small-Cell Lung Cancer	0.33 (2/6)	0.44 (4/9)	0.44 (4/9)	0.44 (4/9)	0.56 (5/9)
	Total Performance	0.5 (80/161)	0.82 (197/239)	0.76 (183/241)	0.7 (164/234)	0.78 (197/251)
BENIGN	Granuloma	0.46 (6/13)	0.96 (24/25)	0.77 (20/26)	0.77 (20/26)	0.88 (23/26)
	Hamartoma	0.5 (3/6)	0.86 (12/14)	0.75 (9/12)	0.92 (12/13)	0.92 (12/13)
	Fibrosis/Scarring/Inflammation	0.5 (12/24)	0.72 (33/46)	0.74 (34/46)	0.76 (31/41)	0.76 (34/45)
	Infection/Pneumonia	0.33 (1/3)	1 (3/3)	1 (3/3)	1 (3/3)	1 (4/4)
	Other Non-Malignant Nodule	0.17 (1/6)	0.86 (6/7)	0.83 (5/6)	0.83 (5/6)	0.71 (5/7)
	Total Performance	0.44 (23/52)	0.82 (78/95)	0.76 (71/93)	0.8 (71/89)	0.82 (78/95)
CONTROL	Stable or Resolving	0.73 (72/99)	0.52 (71/137)	0.5 (72/143)	0.59 (86/145)	0.59 (86/147)
	Interval Increase in Size	0.25 (1/4)	0.33 (2/6)	0.83 (5/6)	0.5 (3/6)	0.67 (4/6)
	New Nodule/Unknown Growth	0.66 (103/156)	0.5 (113/228)	0.6 (144/239)	0.65 (156/241)	0.66 (159/242)
	Mix of New and Stable Nodules	1 (1/1)	1 (1/1)	1 (1/1)	0 (0/1)	1 (1/1)
	No Finding	0.7 (21/30)	0.27 (13/48)	0.56 (24/43)	0.59 (29/49)	0.57 (26/46)
	Total Performance	0.68 (198/290)	0.48 (200/420)	0.57 (246/432)	0.62 (274/442)	0.62 (276/442)

**Table A4:** In Table A1, we show the breakdown of pathological tumor stage for the malignancy cases comprising the Training, Validation 1, and Validation 2 cohorts. Not Available refers to the availability of the staging category within the patient’s medical information uploaded to Epic.

	Training Cohort			Validation 1 Cohort			Validation 2 (Testing) Cohort		
	Total	Non-Actionable	Actionable	Total	Non-Actionable	Actionable	Total	Non-Actionable	Actionable
	*n =* 565	*n =* 260	*n =* 305	*n =* 93	*n =* 48	*n =* 45	*n =* 183	*n =* 84	*n =* 99
Gender									
Male (%)	242 (42.8%)	105 (40.4%)	137 (44.9%)	38 (40.9%)	22 (45.8%)	16 (35.6%)	73 (40.0%)	34 (40.5%)	39 (39.3%)
Age, years Median	67	64	69	67	63	71	67	65	68
Minimum	41	48	41	47	47	51	44	53	44
Maximum	87	82	87	83	77	83	88	82	88
Diagnosis									
NSCLC	159	1	158	31	0	31	55	0	55
AdCa		1	103		0	21		0	36
SqCC		0	47		0	9		0	17
AdCa/SqCC Mixed		0	7		0	1		0	1
NSCLC (Not Specified)		0	1		0	0		0	1
Malignancy, non-NSCLC	22	1	21	2	0	2	5	0	5
Carcinoid (G1/G2)		0	6		0	1		0	1
Large-Cell/SCLC (G3)		0	9		0	1		0	2
Metastasis (Not Lung Cancer)		1	6		0	0		0	2
Benign	73	6	67	6	2	4	22	3	19
Granuloma		1	21		0	1		1	5
Hamartoma		0	12		0	0		0	2
Fibrosis/Scar/Inflammation		5	25		2	2		2	10
Infection/Org. Pneumonia		0	4		0	0		0	1
Other		0	5		0	1		0	1
Not Assessed *	311	252	59	54	46	8	101	81	20
Stable or Resolving		74	18		17	5		32	8
Interval Increase in Size		2	3		0	0		0	1
New Nodule/Unknown Growth		143	36		20	3		39	11
Mix of New/Stable Nodules		1	0		0	0		0	0
No Nodule/Non-Specified		32	2		9	0		10	0

* Patient did not undergo surgery, and thus, results are based on radiological presentation.
